# A Two-step Strategy for High-Value-Added Utilization of Rapeseed Meal by Concurrent Improvement of Phenolic Extraction and Protein Conversion for Microbial Iturin A Production

**DOI:** 10.3389/fbioe.2021.735714

**Published:** 2021-11-17

**Authors:** Meng Wang, Chen Yang, Jean Marie François, Xia Wan, Qianchun Deng, Danyang Feng, Shiyu Deng, Shouwen Chen, Fenghong Huang, Wenchao Chen, Yangmin Gong

**Affiliations:** ^1^ Oil Crops Research Institute of the Chinese Academy of Agricultural Sciences, Wuhan, China; ^2^ Key Laboratory of Biology and Genetic Improvement of Oil Crops, Ministry of Agriculture, Wuhan, China; ^3^ Oil Crops and Lipids Process Technology National and Local Joint Engineering Laboratory, Wuhan, China; ^4^ Hubei Key Laboratory of Lipid Chemistry and Nutrition, Wuhan, China; ^5^ Toulouse Biotechnology Institute (TBI), Toulouse, France; ^6^ Hubei Collaborative Innovation Center for Green Transformation of Bio-Resources, Environmental Microbial Technology Center of Hubei Province, College of Life Sciences, Hubei University, Wuhan, China

**Keywords:** rapeseed meal, two-step strategy, fungal pretreatment, phenolic compounds, protein residue, microbial iturin A production

## Abstract

Rapeseed meal (RSM) is a major by-product of oil extraction from rapeseed, consists mainly of proteins and phenolic compounds. The use of RSM as protein feedstock for microbial fermentation is always hampered by phenolic compounds, which have antioxidant property with health-promoting benefits but inhibit bacterial growth. However, there is still not any good process that simultaneously improve extraction efficiency of phenolic compounds with conversion efficiency of protein residue into microbial production. Here we established a two-step strategy including fungal pretreatment followed by extraction of phenolic compounds. This could not only increase extraction efficiency and antioxidant property of phenolic compounds by about 2-fold, but also improve conversion efficiency of protein residue into iturin A production by *Bacillus amyloliquefaciens* CX-20 by about 33%. The antioxidant and antibacterial activities of phenolic extracts were influenced by both total phenolic content and profile, while microbial feedstock value of residue was greatly improved because protein content was increased by ∼5% and phenolic content was decreased by ∼60%. Moreover, this two-step process resulted in isolating more proteins from RSM, bringing iturin A production to 1.95 g/L. In conclusion, high-value-added and graded utilization of phenolic extract and protein residue from RSM with zero waste is realized by a two-step strategy, which combines both benefits of fungal pretreatment and phenolic extraction procedures.

## Introduction

Rapeseed is a valuable crop mainly used in the production of edible oil and animal feed (Hussain et al., 2019). Over 70 millions tons are harvested yearly from which 2.1 tons of rapeseed meal (RSM) constituted a potential upgradeable by-product obtained from each ton of edible oil (Morosanu et al., 2017). Rapeseed is also reported as containing phenol-based antioxidant with the majority of them remaining in RSM after oil extraction (Quinn et al., 2017). In the recent years, RSM has mainly been applied in two aspects: i) use as protein source for animal feed or microbial fermentation feedstock (García et al., 2013; Uçkun Kiran et al., 2013; Shi et al., 2015; Konkol et al., 2019), and ii) extraction of phenolic compounds and exploiting its antioxidant property on food and pharmaceutical (Li and Guo, 2016; Quinn et al., 2017; Hussain et al., 2019; Nandasiri et al., 2019).

In recent years, the recovery of bioactive phenolic compounds present in agricultural by-products such as RSM has been highlighted due to their biological activity, which are associated with health-promoting benefits such as modify various diseases (including cancer, diabetes and cardiovascular diseases), hepatoprotective, anti-obesity, anti-mutagenic, anti-allergenic, anti-inflammatory, and anti-microbial effects (Kim and Lee, 2017; Pohl et al., 2018; Hussain et al., 2019; Nandasiri et al., 2019; Teles et al., 2019; Yates et al., 2019). Sinapic acid and sinapine, as the predominant phenolic compounds in RSM, were also widely studied. Sinapic acid has been shown to possess histone deacetylase (HDAC) and angiotensin-I converting enzyme (ACE-I) inhibitory activity, which were known to play a protective role against diabetes and cardiovascular disease development (Quinn et al., 2017). Sinapine was a acetylcholine esterase (AChE) inhibitor for many diseases including Alzheimer and muscle disease (Nićiforović and Abramovič, 2014). Hepatoprotective or neuroprotective properties of sinapine were also reported in CCl_4_-induced hepatotoxicity mice models or in a PC12 (rat pheochromocytoma) hypoxia cell model from Na_2_S_2_O_4_-induced apoptosis and mitochondrial transmembrane potential disruption (Fu et al., 2016; Yates et al., 2019). Moreover, sinapic acid and sinapine were reported to be capable of delaying oxidation of cellular components (proteins, lipids, and DNA), which contributed to extend their application as food and pharmaceutical additives beneficial for human health or in food preserving measurements (Kim and Lee, 2017; Hussain et al., 2019; Nandasiri et al., 2019; Yates et al., 2019).

However, the anti-nutritional and antibacterial properties of phenolic compounds are the principal bottleneck in using RSM as animal feed and microbial feedstock (García et al., 2013; Uçkun Kiran et al., 2013; Shi et al., 2015; Kim and Lee, 2017). Therein, the presence of phenolic compounds in the rapeseed by-products is a hindrance in its use as a protein fermentation substrate because it caused a strong inhibition of bacterial activity. As for instance, it was reported that NaOH treated-rapeseed cake extract showed potent antimicrobial activity against selected Gram-positive and Gram-negative bacteria, such as *Bacillus*, which was explained by a high content of phenolic and sinapic acid (Kim and Lee, 2017). We also previously showed that fungal pretreatment of RSM had an unconventional inhibitory effects on iturin A production by *B. amyloliquefaciens* CX-20 compared with direct bio-utilization of RSM as the nitrogen source. We argued that this inhibitory effect was due to the presence of compounds such as phenolics with antibacterial effect released by fungal pretreatment (Chen et al., 2021). Therefore, removing as much as possible these antibacterial compounds from RSM should improve its exploitation as valuable protein feedstock substrate for production of iturin A by microbial fermentation. While an efficient extraction of phenolic compounds from RSM could lead to a functional product in food and pharmaceutical industries, the removal of these anti-nutritional and antibacterial compounds should enhance the value of remaining meal or protein residue for microbial feedstock if the protein content in this extraction procedure is not lost. Up to date, there is still not any good process aiming at solving this issue for RSM.

Fungal pretreatment by solid-state fermentation (SSF) is a potent, cost-effective and environmentally friendly tool for the valorization of agricultural by-products such as RSM. It can be used i) to improve the chemical composition and physicochemical properties of the material for animal feed, especially as protein source (Shi et al., 2015; Shi et al., 2016; Wang et al., 2020), ii) to release nutrients for value-added microbial products, especially as nitrogen and carbon source (García et al., 2013; Uçkun Kiran et al., 2013; Chatzifragkou et al., 2014; Salakkam et al., 2017; Salakkam and Webb, 2018), and iii) to facilitate the extraction of bioactive compounds, especially antioxidant phenolic compounds (Martins et al., 2011; Schmidt et al., 2014; Bhanja Dey et al., 2016). In this study, we explored whether a two-step method, combining fungal pretreatment followed by extraction of phenolic components, is a feasible strategy for improving the microbial feedstock value of RSM as a protein source for iturin A production. *Aspergillus oryzae* 92,011 and *Trametes* sp. 48,424 were selected for fungal source to carry out these pretreatments, due to their ability to produce various glycosylhydrolase enzymes that degrade plant cell wall (Chen et al., 2021). Following this first step, we examined whether ethanol under acidic condition could be an efficient extraction condition to extract as much as antioxidant phenolic compounds from RSM while minimize protein loss. The biochemical composition of the remaining meal or protein residue treated using this two-step process was extensively analyzed and investigated for their potential as raw substrate for iturin A production by *B. amyloliquefaciens* CX-20 (Supplementary Figure S1).

## Materials and Methods

### Microorganisms, Media and Fungal Pretreatment

The strain *B. amyloliquefaciens* CX-20 (CCTCC NO: M 2018794) was kindly provided by Professor Shouwen Chen (College of Life Sciences, Hubei University, Wuhan, China). Seed cultures were carried out in Luria-Bertani (LB) medium (10 g tryptone, 5 g yeast extract, and 10 g NaCl per litre). Unless otherwise stated, the fermentation medium was composed of (per liter) 80 g glucose, inorganic salts (1 g K_2_HPO_4_·3H_2_O, 0.5 g MgSO_4_ 7H_2_O, 0.005 g MnSO_4_·H_2_O) and 90 g RSM. The initial pH of the medium was adjusted to 7.0, followed by sterilization at 121°C for 30 min. Flask experiments were performed in 250 ml flasks with a fermentation volume of 20 ml and an inoculation size of 5% (v/v). All fermentations were carried out at 28°C for 72°h, under constant orbital shaking at 220 rpm.

*A. oryzae* 92,011 (CCTCC No: AF 92011) was kindly provided by the China Center for Type Culture Collection (Wuhan). *Trametes* sp. 48,424 was obtained from the School of Life Science and Technology, Huazhong University of Science and Technology, Wuhan, China (Chen et al., 2021). The strains were maintained in the form of spores, in dry sand at 4°C. Prior to each SSF, two fungi were sporulated on the surface of solid medium containing 25 g/L RSM, 25 g/L wheat bran and 20 g/L agar, in 20 ml test tubes incubated for 5°days at 28°C and resuspended in 10°ml of sterile distilled water with 0.01% Tween 80 (v/v) to each test tube. These spore suspensions served as inocula for SSF, which was performed in pre-sterilized (121°C for 30 min) 5 L flasks containing 1°kg of RSM as the sole source of nutrients. The moisture content was adjusted to 65% (w/w) after inoculation with fungal spores. All flasks were seeded with approximately 10^6^ spores/g RSM and incubated at 28°C for 72 h.

### Extraction Procedure of Antioxidant Compounds

Acidic ethanol is the most efficient solvent for recovering the soluble phenolics from RSM and simultaneously minimizing protein loss (Liu et al., 2012; Kim and Lee, 2017; Hussain et al., 2019; Nandasiri et al., 2019). RSM has strong water-binding capacity, so less solution would be obtained when solid-liquid ratio reached 1:2 (Pustjens et al., 2012). Hence, 1:4 solid-liquid ratio was used for large scale melanin extraction to save cost. Untreated and fungal treated RSMs were mixed with 70% ethanol solution containing 0.5 mol/L HCl at ratio of 1:4. The mixture was shaken at 150 rpm at 40°C for 1°h, followed by centrifugation at 4,000 rpm for 10 min. The obtained supernatant was poured on a AB-8 macroporous resin (HaoJu resin Technology Co. Ltd., Tianjin, China) and then eluted with a standard 70% ethanol solution. The ethanolic fractions were dried with a vacuum evaporator to obtain the bioactive compound extracts by resin adsorption. The extracts derived from RSM, RSM pretreated by *A. oryzae* 92,011 and *Trametes* sp. 48,424 were designated as RSME, 92011E and 48424E, respectively.

### Extra-Protein Isolation Using Alkaline Extraction of Rapeseed Meal Residues

The residues after ethanolic extraction were designated as RSMeR, 92011eR and 48424eR, which were derived from unpretreated RSM, RSM pretreated by *A. oryzae* 92,011 and *Trametes* sp. 48,424, respectively. After drying, the residues were ground to pass through a 120-mesh screen and then dispersed in deionized water (1:12 w/v), adjusted to pH 12.0 with 1 M sodium hydroxide and stirred at 55°C for 40 min. The slurry was centrifuged at 4,000 rpm for 10°min, the supernatant recovered, and the meal re-extracted twice (1:10 w/v and 1:8 w/v, respectively) as described above. The resulting supernatants were combined, adjusted to pH 4.5 with 1 M citric acid and centrifuged at 4,000 rpm for 10 min again. The obtained pellet were rinsed once with water and lyophilized for further use. Isolated protein by alkaline extraction from RSM, RSMeR, 92011eR or 48424eR was designated as RSM-P, RSMeR-P, 92011eR-P or 48424eR-P, respectively.

### Metabolomic Analysis

A 5 mg RSME, 92011E or 48424E were added to 100 µL of ice-cold methanol and vortexed for 1 min with subsequent ultrasound for 5 min. The mixture was kept at −20°C for 2 h, and then centrifuged at 14,000 rpm at 4°C for 20 min to precipitate the proteins. The protein free supernatant was collected and dried with nitrogen at 37°C. The concentration of RSME, 92011E or 48424E was diluted depending on the mass spectrum response signals.

Chromatographic separation was performed on an ACQUITY UPLC HSS T3 column (100 mm×2.1 mm, 1.8 µm, Waters, UK) using a 2777 C UPLC system (Waters). The flow rate was 0.40 ml/min, and the mobile phase was composed of solvent A (water+ 0.1% formic acid) and solvent B (acetonitrile + 0.1% formic acid). The gradient program was optimized as follows: 0–2 min, 100% phase A; 2–11 min, 0–100% B; 11–13 min, 100% B; 13–15 min, 0–100% A. The injection volume was 10 μL. The eluent from the column was added directly to the mass spectrometer without splitting. Mass spectrometry with an electrospray ionization source (ESI) operating in positive and negative ion mode was carried out using a Xevo G2-XS QTOF (Waters, UK) mass spectrometer. The capillary voltages were set at 3.0 kV (+) and 2.0 kV (−), and the sample cone voltage was 40 V. The mass spectrometry data were acquired in Centroid MSE mode. The TOF mass range was from 50 to 1,200 Da and the scan time was 0.2 s. For the MS/MS detection, all precursors were fragmented using 20–40 eV, and the scan time was 0.2 s. During the acquisition, the LE signal was acquired every 3 s to calibrate the mass accuracy. Furthermore, in order to evaluate the stability of the LC-MS during the whole acquisition, a quality control sample (Pool of all samples) was acquired after every 10 samples.

### Inhibitory Effects of Ethanolic Extracts on Iturin A Production

According to the previous study (Chen et al., 2019a), the initial optimal RSM-P concentrations for iturin A production by *B. amyloliquefaciens* CX-20 was 50 g/L. Therefore, in order to evaluate inhibitory effects of ethanolic extracts (RSME, 92011E and 48424E) on iturin A production, the fermentation medium, composed of (per liter) 80 g glucose, inorganic salts (1 g K_2_HPO_4_·3H_2_O, 0.5 g MgSO_4_·7H_2_O, 0.005 g MnSO_4_·H_2_O) and 50 g RSM-P, was taken as the control. Different initial ethanolic extract concentrations ranging from 0 to 8 g/L were added into above medium. The initial pH of the medium was adjusted to 7.0, followed by sterilization at 121°C for 30 min. Flask experiments were performed in 250 ml flasks with a fermentation volume of 20 ml and an inoculation size of 5% (v/v). All fermentations were carried out at 28°C for 72 h, under constant orbital shaking at 220 rpm.

### Improvement of Protein Utilization by Two-step Strategy for Iturin A Production

The fermentation medium, composed of (per liter) 80 g glucose, inorganic salts (1 g K_2_HPO_4_·3H_2_O, 0.5 g MgSO_4_·7H_2_O, 0.005 g MnSO_4_·H_2_O) and 50 g RSM-P, was taken as the control. In order to evaluate the improvement of RSM conversion by two-step strategy for iturin A production, 50 g RSM-P was substituted by different initial concentrations of RSMeR-P, 92011eR-P or 48424eR-P ranging from 30 to 90 g/L, respectively. The initial pH of the medium was adjusted to 7.0, followed by sterilization at 121°C for 30 min. Flask experiments were performed in 250 ml flasks with a fermentation volume of 20 ml and an inoculation size of 5% (v/v). All fermentations were carried out at 28°C for 72 h, under constant orbital shaking at 220 rpm.

### Analytical Methods

The fermented RSM and untreated RSM samples were analyzed for dry matter, crude protein, crude fiber and glucosinolates content (Shi et al., 2016). The total phenolic content was determined using the Folin−Ciocalteu assay, according to the method described previously by Quinn et al. (2017). The radical DPPH (2, 2-Diphenyl-1-picrylhydrazyl) scavenging activity of the samples was measured using the method by Pohl et al. (2018) with minor modifications. Serial dilutions of all phenolic extracts were prepared in methanol and 50 μL mixed with freshly prepared DPPH solution (100 μL; 0.75 mM in methanol), to yield final extract concentrations between 5 and 500 μg/ml. The plates were incubated in the dark (30 min; at room temperature) and the absorbance was read at 517 nm. Furthermore, a serial dilution (5–500 μg/ml) of SA was analysed for comparison. The linear part of the obtained curve was used to determine the IC 50 value, by plotting a linear graph and using the trend line for IC 50 calculations.

Iturin A was determined by high-performance liquid chromatography (HPLC) according to a reported method by Chen et al. (2019a). The concentrations of reducing sugar were determined using the DNS method (Miller, 1959). The viable cell count during submerged fermentation was determined as follows: 0.5 ml of the sample was placed into a sterile 10 ml test tube, mixed thoroughly with 4.5 ml of sterile distilled water and shaken at 150 rpm on a vortex for 5 min at room temperature. The mixture was then serially diluted and spread onto LB agar plates. After 24 h of incubation at 28°C, the number of colonies was counted and expressed as colony forming units (CFU).

All experiments were performed in triplicate and the data were processed using GraphPad Prism 8.0.1 and Adobe Illustrator CC.

## Results and Discussion

### Analysis of the Characteristics of the Phenolic Extracts Obtained by the Two-Step Process

**FIGURE 1:**
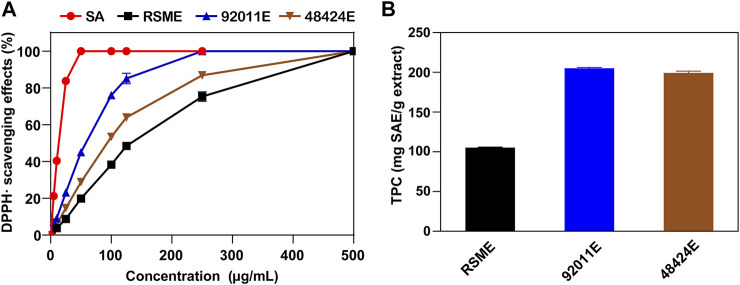
Scavenging effects on DPPH radicals **(A)** and TPC **(B)** of three phenolic extracts.

RSM was firstly subjected to fungal pretreatment using either *A. oryzae* 92,011 or *Trametes* sp. 48,424, and the resulting product was designated as 92011F or 48424F. The second step was the extraction of antimicrobial phenolic compounds from 92011F or 48424F by 70% ethanol in acidic condition. The corresponding solid extract obtained after this two step process was designated as 92011E or 48424E, while the corresponding dried residue was designated as 92011eR or 48424eR. The solid extract and dried residue termed RSME and RSMeR were also prepared from RSM but the fungal pretreatment was skipped (Supplementary Figure S1). Using DPPH as the scavenger of free radical, antioxidant activity was evaluated in all of these extracts. As shown in Figure 1A, the IC_50_ value of 92011E and 48424E was 2.13 and 1.49 fold lower than for RSME (66.59 and 94.95 μg/ml versus 141.56 μg/ml), suggesting that the extracts had been enriched with antioxidant compounds upon pretreatment of the RSM with fungi. Direct quantification of total phenolic contents (TPC) in the three extracts by Folin-Ciocalteu (FC) supported the DPPH data as it was found that TPC values in 92011E and 48424E were about 2-fold higher than in RSME [205.01 ± 1.11 and 199.36 ± 2.14 mg SAE (Sinapic acid equivalents)/g versus 105.31 ± 0.73 mg SAE/g] (Figure 1B). However, effects of fungal pretreatment *per se* on phenolic profile, as another potential factor to alter antioxidant property, could not be excluded.

**FIGURE 2:**
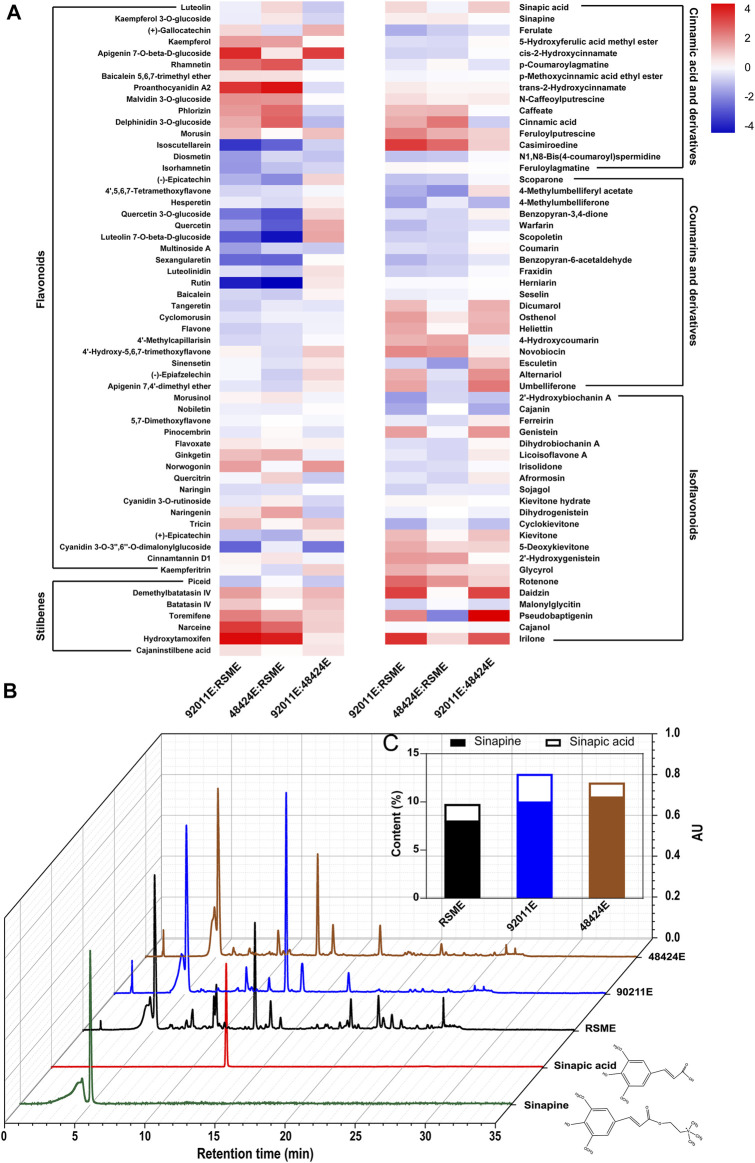
Effects of fungal pretreatment on phenolic profile. Heat maps of phenolic profile differences among three phenolic compound extracts **(A)**, comparison of the UPLC chromatograms of three phenolic extracts **(B)** and sinapic acid and sinapine contents in three bioactive compound extracts **(C)**.

Metabolomics analysis using LC-MS was employed to identify and quantify the various antioxidants compounds that were present in all different extracts from RSM. Lists of all the compounds that were identified were reported in Supplementary material, which included 50 flavonoids (Supplementary Table S1), 15 cinnamic acids and derivatives (Supplementary Table S2), 19 coumarins and derivatives (Supplementary Table S3), 22 isoflavonoids (Supplementary Table S4) and 7 stilbenes (Supplementary Table S5). A heat map, based on the ratio of 113 phenolic compounds of 92011E versus RSME, 48424E versus RSME and 92011E versus 48424E, was built to facilitate the comparison of the profile of the phenolic compounds between these three extracts (Figure 2A). Epicatechin, isorhamnetin, kaempferol, quercetin, and their derivatives that are the major flavonoids in *Brassicaceae* plants (Brito et al., 2020; Qu et al., 2020) were also identified in RSM extracts. Except for kaempferol, the contents of epicatechin, isorhamnetin and quercetin were reduced respectively by more than 73, 67 and 80% by our two-step strategy (the average value of the ratio of 92011E versus RSME and 48424E versus RSME). Notably, proanthocyanidin, which was a type of prominent flavonoid compound deposited in seed coats and which controls the pigmentation of *Brassica* species (Liu et al., 2016), increased more than 15- and 24-fold in 92011E and 48424E, respectively. This might explain why the dark color of residues (92011eR or 48424eR) after two-step strategy became significantly lighter than RSM. The content of cinnamic acid was increased about 3- and 6-fold in 92011E and 48424E, respectively. In accordance with previous works (Kim and Lee, 2017; Pohl et al., 2018; Hussain et al., 2019; Nandasiri et al., 2019; Yates et al., 2019), sinapic acid and sinapine were the predominant phenolic compounds identified in three ethanolic extracts of RSM (Figure 2B). However, fungal pretreatment increased the total content of sinapine and sinapic acid by 32% in 92011E and 23% in 48424E as compared to RSME (Figure 2C). In this study, the increased content of TPC and some phenolics (such as sinapic acid, sinapine, kaempferol, proanthocyanidin and cinnamic acid) by fungal pretreatment showed that two-step strategy facilitated the extraction process, making it an ideal method of extraction for studying the structure-function relationship of phenolic compounds. In summary, compared to the sole ethanolic extraction process, our two-step strategy was more efficient at extracting more antioxidant phenolic compounds from RSM. The corresponding higher antioxidant activities of extracts (92011E and 48424E) by two-step strategy were influenced by both TPC and phenolic profile.

Although fungal pretreatment has been widely used to improve the extraction of phenolic compounds from agricultural by-products (Martins et al., 2011; Schmidt et al., 2014; Bhanja Dey et al., 2016), it was first attempted for RSM in this study. Different phenolic profile produced by different fungal pretreatment might be related to the two enzymatic cocktails produced by *A. oryzae* 92,011 or *Trametes* sp. 48,424, respectively, which was consistent with the report by Teles et al. (2019). However, there are limitations to this work. The phenolic extract isolated as part of this work is not purified. Corresponding functions and potential applications needed further study.

### Inhibitory Effects of Ethanolic Extracts From Rapeseed Meal on Iturin A Production by *B. amyloliquefaciens* CX-20

**FIGURE 3:**
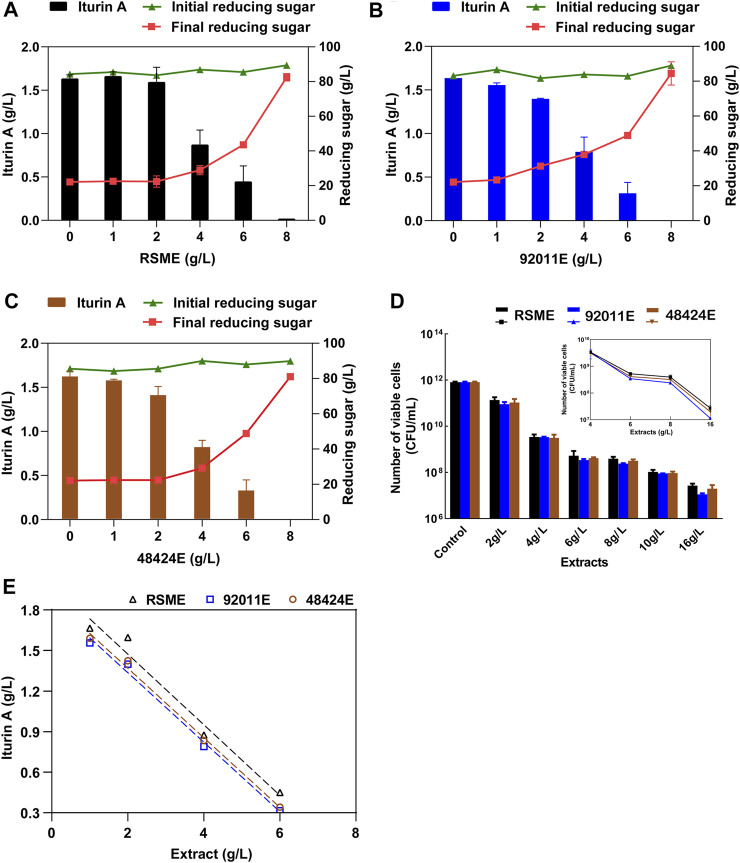
Inhibitor effects of phenolic extracts from RSM on iturin A production by *B. amyloliquefaciens*. **(A)** Effects of different concentrations of RSME on iturin A production, concentrations of initial and final reducing sugars. **(B)** Effects of different concentrations of 92011E on iturin A production, concentrations of initial and final reducing sugars. **(C)** Effects of different concentrations of 48424E on iturin A production, concentrations of initial and final reducing sugars. **(D)** Effects of different concentrations of three phenolic extracts on the maximum number of viable cells of *B. amyloliquefaciens* CX-20. **(E)** The negative linear relationships between the iturin A production and additive amount of RSME, 92011E and 48424E.

As expected, the addition of RSME, which was obtained from direct ethanol extraction of RSM to the fermentation medium, caused a strong reduction of the iturin A production by *B. amyloliquefaciens* CX-20. This reduction was proportional to the amount of RSME with a complete arrest of production at 8 g/L of RSME added (Figure 3A). A very similar inhibitory effect of iturin A production was obtained with 92011E and 48424E which were obtained by our two-step strategy (Figures 3B,C). However, we noticed that inhibitory effects by these extracts were slight stronger than that of RSME in the range of 1–4 g/L. It was reported that the antibacterial effects of phenolic extracts were positively correlated with the antioxidant activities and TPC (Gottardi et al., 2016). However, the correlation was weak in this study, as the antioxidant activity of 92011E and 48424E was about twice than RSME while the inhibitory effects of three ethanolic extracts on iturin A production and viable cell count of *B. amyloliquefaciens* CX-20 were not significantly different (Figures 3D,E). These suggested that phenolic antioxidants present in RSM were not the sole compounds to account for this inhibition of iturin A production by *Bacillus* fermentation of RSM, such as reported glucosinolates and phospholipids (Li and Guo, 2016; Pohl et al., 2018).

Since the consumption of reducing sugar also decreased with the increase of extracts added to the medium, we verified whether this inhibition of iturin A production was due to arrest of bacterial fermentation or loss of cell viability. Figure 3D showed that the cell viability dropped proportionally with the increase of ethanolic extract, whatsoever its origin and at 8 g/L where the production iturin A by *B amyloliquefaciens* CX-20 was completely stopped, there was less than 0.05% of viable bacterial cells. This result indicated that the inhibition of iturin A production was most probably due to the loss of viability of the cell caused by the toxicity of the ethanolic extracts, which was consistent with the previous report (Chen et al., 2019b). Phenolic compounds are found in plants as defense mechanisms and always have antimicrobial activity. Until now, sinapic acid in RSM was thought to be the main compound for antibacterial effects against spoilage bacteria and yeast such as *Staphylococcus aureus*, *Listeria monocytogenes*, *Escherichia coli*, *Lactobacillus plantarum*, *Bacillus subtilis* and *Candida albicans* (Kim and Lee, 2017). It was reported that sinapic acid could diffuse across microbial cell membranes as an undissociated acid and cause acidification of the cytoplasm and eventually cell death. Structurally, sinapic acid had 2 methoxyl groups and a hydroxyl group on a benzene ring, as well as double-bonded side chain, which contributed to its antibacterial effects (Sánchez-Maldonado et al., 2011). Although sinapine in rapeseed was reported to have no antibacterial effects, it could be converted into sinapic acid by alkali or enzymatic hydrolysis (Li and Guo, 2016; Kim and Lee, 2017). The possible synergistic effects of sinapic acid and sinapine or other antioxidant compounds on antimicrobial activity are still unclear. This antimicrobial activity of RSM ethanolic extract is a double-edged sword. On the one hand, it can be used as food additive for supporting microbial stability during storage (Kim and Lee, 2017). On the other hand, this property might suppress the microbial conversion efficiency of RSM for producing high-value-added metabolites. The key is how to take advantage of this feature.

### Effects of Two-Step Strategy on the Composition of Extraction Residues and Extra Protein Isolation

**FIGURE 4:**
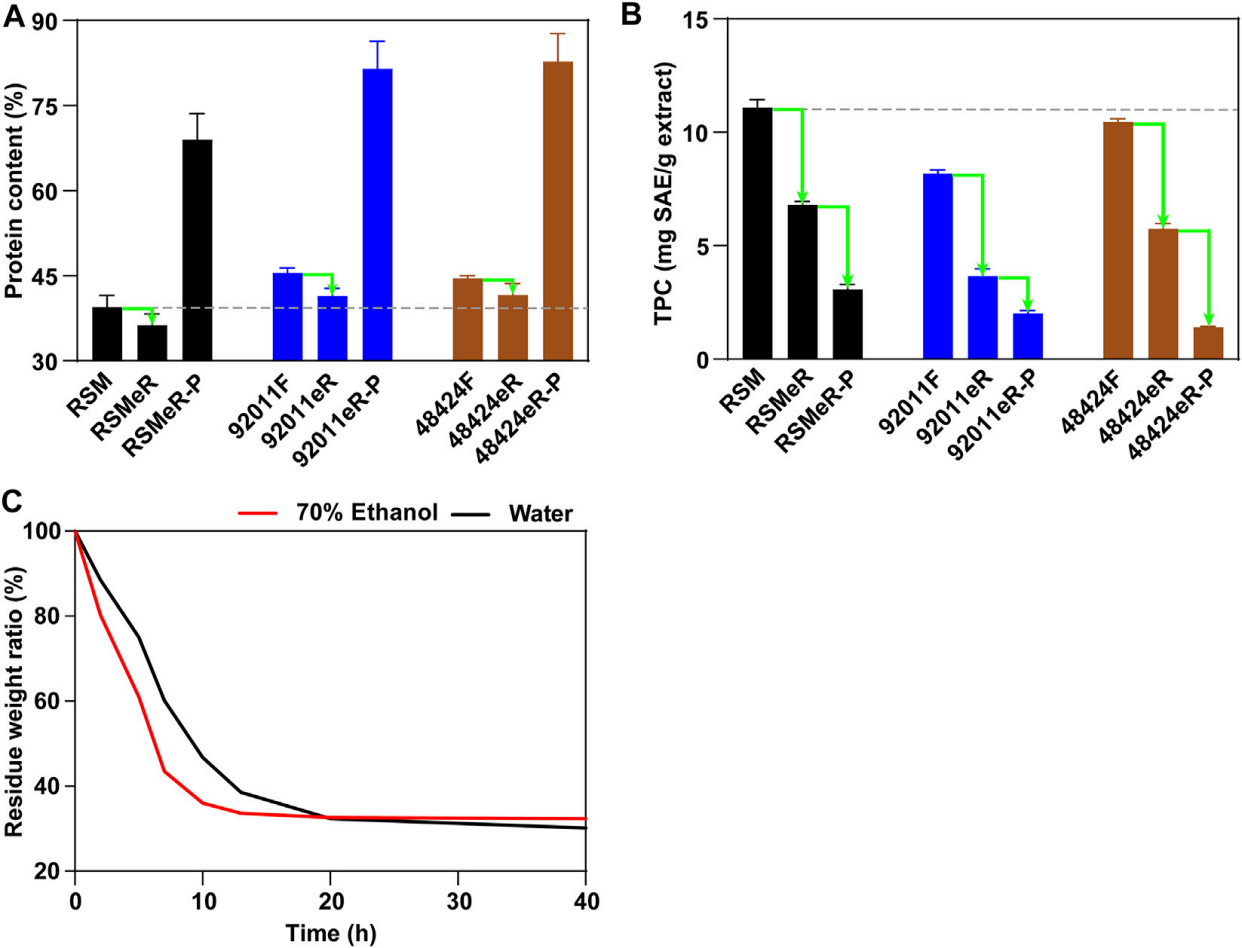
Effects of two-step strategy and extra protein extraction on the protein content **(A)** and the TPC **(B)**, and the advantage of ethanol extraction for drying residue of RSM **(C)**.

To verify that our two-step strategy was effective in improving feedstock quality of RSM, the composition of remaining meal or residue was analyzed and compared between each other. Pretreatment with *A. oryzae* 92,011 and *Trametes* sp. 48,424 increased the proteins content in 92011F and 48424F by about 13% as compared to untreated RSM (from 39.48 to 45.51% and 44.54%, respectively), mainly due to the decreased carbohydrate content after fermentation (Chen et al., 2021). The extraction residues derived from untreated RSM, 92011F and 48424F were designated as RSMeR, 92011eR and 48424eR. Although the subsequent ethanolic extraction step decreased the protein content in 92011eR and 48424eR by about 4%, the total contents of protein in 92011eR and 48424eR were still about 5% higher than in RSM (Figure 4A). It was reported that alkaline extraction is a good procedure that allow good isolation of proteins from RSM (Chen et al., 2019a). In this study, isolated protein by alkaline extraction from RSMeR, 92011eR and 48424eR were designated as RSMeR-P, 92011eR-P and 48424eR-P. Our results also demonstrated that extra protein isolation could further improve the protein content from three residues. However, two-step strategy had obvious advantages so that the protein content in RSMeR-P only increased to 69.00%, while that in 92011eR-P and 48424eR-P could reach 81.55 and 82.76%, respectively. This was consistent with the report by Rodrigues et al. (2017) and indicated that pretreatment with enzymes secreted by fungi could increase the yield of protein extraction. As Table 1 shown, beside the increase of protein content, two-step strategy could also reduced the concentration of anti-nutritional factors, which resulted in poor digestibility, unpleasant color and bitter taste for animal feed (Hernández-Jabalera et al., 2015; Li and Guo, 2016; Quinn et al., 2017; Hussain et al., 2019; Nandasiri et al., 2019). Especially for glucosinolates and sinapine, the content respectively decreased by more than 76 and 67% in 92011eR compared with RSM. This higher removal of anti-nutritional should be beneficial for direct utilization of the protein residues from two-step strategy as animal feed.

**TABLE 1:** Effects of fungal pretreatment and antioxidant extraction on the composition of RSM.

	RSM 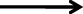	RSMeR	92011F 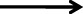	92011eR	48424F 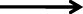	48424eR
Dry matter (%)	90.23	95.17	94.21	95.32	94.66	94.93
Protein (%)	39.48	36.30	45.51	41.42	44.54	41.62
Glucosinolates (μmol/g)	13.38	4.38	3.94	3.17	15.39	5.18
Tannin (g/kg)	0.11	0.06	0.30	0.09	0.94	0.49
Crude Fiber (%)	12.02	12.32	10.14	10.11	10.14	9.76
Sinapine (%)	0.98	0.68	0.39	0.32	0.81	0.61
Sinapic acid (%)	0.03	0.02	0.15	0.08	0.08	0.05

Note: Fermented RSM by *A. oryzae* 92,011 or *Trametes* sp. 48,424 was designated as 92011F or 48424F, while the extract residues derived from unpretreated RSM, 92011F and 48424F were designated as RSMeR, 92011eR and 48424eR, respectively.

In addition, our two-step strategy simultaneously reduced the levels of TPC by about 67% in 92011eR (from 11.09 ± 0.36 to 3.66 ± 0.32 mg SAE/g) and 48% in 48424eR (from 11.09 ± 0.36 to 5.74 ± 0.24 mg SAE/g), while the TPC in RSMeR was only reduced by 39% (from 11.09 ± 0.36 to 6.80 ± 0.15 mg SAE/g) (Figure 4B). Taking the TPC in three extracts (RSME, 92011E and 48424E) (Figure 1) into consideration, it could be seen that the TPC in corresponding residue was negatively correlated with the TPC in the extract. Moreover, extra protein isolation resulted in a further removal of TPC. The TPC in RSMeR-P, 92011eR-P and 48424eR-P was further reduced to 3.07 ± 0.22, 2.02 ± 0.12 and 1.41 ± 0.04 mg SAE/g, respectively (Figure 4B). In summary, our two-step strategy had the advantages of simultaneously increasing the protein content and decreasing TPC either in ethanolic extraction residues or in extra protein isolates, which is expected to be beneficial for animal feed or microbial fermentation.

Ethanol, which was applied as an efficient and green co-solvent to change the polarity of water and then improve the recovery of antioxidant compounds, especially in rapeseed by-products (Kim and Lee, 2017; Pohl et al., 2018; Hussain et al., 2019; Nandasiri et al., 2019; Yates et al., 2019), was also the chosen solvent for high volume extraction in this study. Moreover, ethanol is more volatile than water, and it took less time and cost to dry the residue. As shown in Figure 4C, only 12 h were needed to dry the residue from 70% ethanol at 60°C, which was much shorter than the 20 h needed for drying the residue from water. All these results further indicated that two-step strategy could improve the value of RSM residue as animal and microbial feed with reduced time and economic cost.

### Improvement of Rapeseed Meal Conversion by Two-step Strategy for Iturin A Production

**FIGURE 5:**
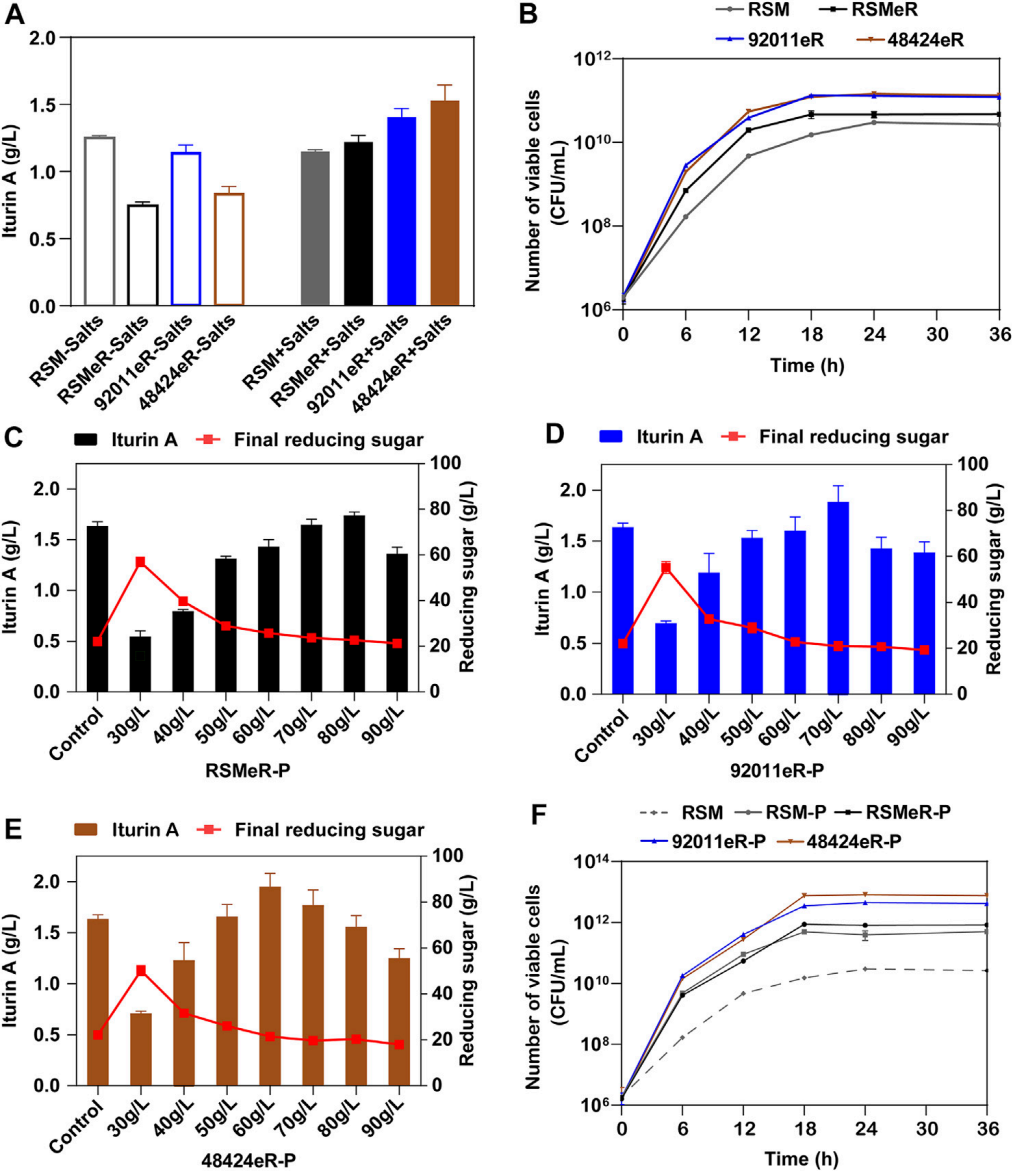
Effects of two-step strategy on residue’s value for iturin A production **(A)** and the growth of *B. amyloliquefaciens* CX-20 **(B)**. -Salts means that not any inorganic salts is added into the medium, while + Salts means that inorganic salts is added into the medium. Effects of different initial concentrations of RSMeR-P **(C)**, 92011eR-P **(D)** and 48424eR-P **(E)** on iturin A production and final concentration of reducing sugar. (F) The effect of optimal concentration of RSM, RSM-P, RSMeR-P, 92011eR-P and 48424eR-P on the growth of *B. amyloliquefaciens* CX-20.

In a previous work, we showed an achievement of 1.25 g/L iturin A after 72 h of *B. amyloquefaciens* fermentation in medium that only contained 80 g/L glucose and 90 g/L RSM (Chen et al., 2019a). Here, the RSM was replaced by either RSMeR, 92011eR or 48424eR. Unexpectedly, we found a reduced production of iturin A. Inorganic salts or mineral elements in RSM, such as K^+^, Mg^2+^, were important factors in microbial fermentation (Chen et al., 2019a) but were likely removed by the ethanolic extraction. Therefore, we completed the medium with inorganic salts, and found not solely a recovery of iturin A production with RSMeR (1.22 g/L) but increases by 15% with 92011eR (1.40 g/L) and 25% with 48424eR (1.53 g/L) (Figure 5A). The corresponding growth curves also demonstrated that the higher production of iturin A was related to higher specific growth rate and higher maximum viable cell count (Figure 5B). Compared with RSM or residue produced by single ethanolic extraction procedure (RSMeR), the residues produced by two-step strategy (92011eR and 48424eR) were more suitable for *Bacillus* fermentation for iturin A synthesis, mainly due to both a higher protein content and lower levels of antibacterial components. The protein enrichment in RSMeR-P, 92011eR-P or 48424eR-P further enhanced the iturin A production, reaching 1.74, 1.88 or 1.95 g/L at their optimal initial concentration which was 80 g/L for RSMeR-P, 70 g/L for 92011eR-P and 60 g/L for 48424eR-P, respectively (Figures 5C–E). As compared with direct utilization of RSM (1.15 g/L), this corresponded to an increase of iturin A production of 51, 64 and 70%, respectively. The corresponding growth curves further verified that iturin A production was positively related to microbial growth (Figure 5F).

**FIGURE 6:**
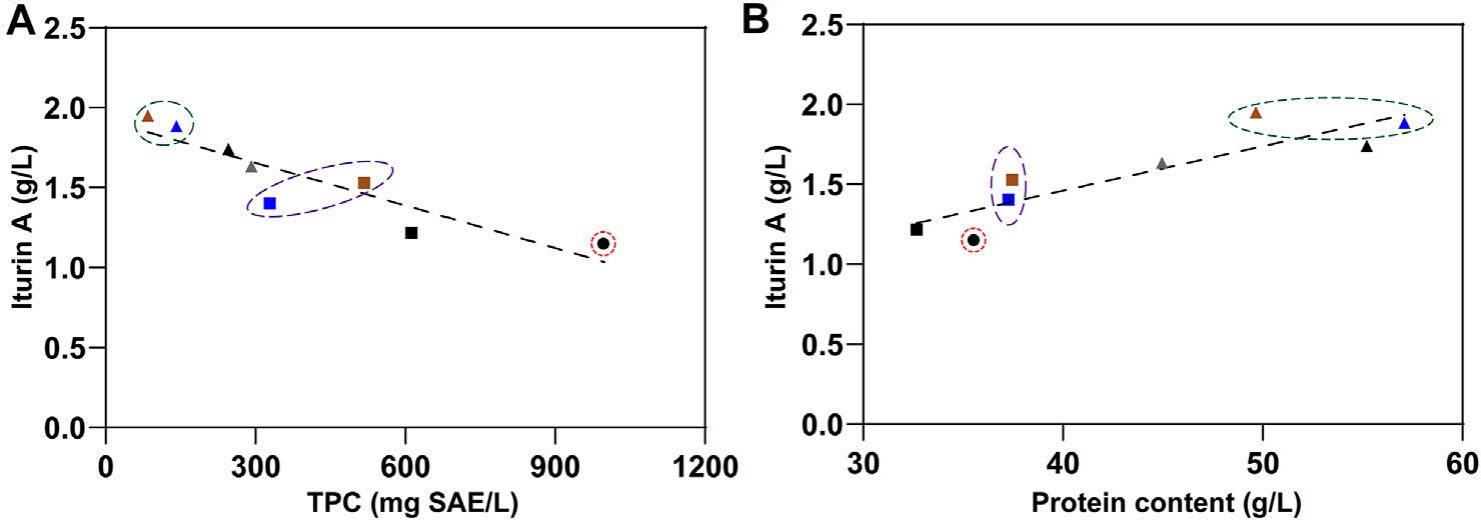
The relationship between TPC and iturin A production **(A)** or between protein content and iturin A production **(B)**. Black circle represented RSM. Black, blue and brown square represent RSMeR, 92011eR and 48424eR, respectively. Gray, black, blue and brown triangle represent RSM-P, RSMeR-P, 92011eR-P and 48424eR-P, respectively.

Although the single fungal pretreatment procedure was beneficial to increase the protein content and to release antioxidant phenolic compounds from RSM likely by degrading or breaking complex matrix compounds such as polysaccharides and lignin (Shi et al., 2015, 2016; Bhanja Dey et al., 2016; Salakkam et al., 2017; Salakkam and Webb, 2018; Zhu et al., 2018; Wang et al., 2020; Chen et al., 2021), the antibacterial property of the obtained treated RSM was still important. At the same time, the single ethanolic extraction procedure has been shown to be effective in removing most of the antibacterial and antioxidant components from RSM, but its drawback was to reduce the protein content present in the final residue. Biorefinery represents the most effective holistic approach to achieving a circular economy using the biotransformation of agricultural biomass with a complex, mixed-component, aiming to produce zero waste (Konkol et al., 2019). This goal can not be achieved by a single technical method. Here, we showed that a two-step strategy made it possible to combine the benefits of these two procedures and overcome both drawbacks. Fungal pretreatment can completely offset the protein loss by ethanolic extraction, while the latter removed the antioxidant and antibacterial phenolic compounds released and increased by the former. Finally, there were two raw substances obtained by our two-step strategy. The first one was the phenolic extract with improved antioxidant and antibacterial property. The second was residues with enhanced value as animal feed and microbial feedstock, which contained more protein and less anti-nutritional and antibacterial components, especially TPC. The correlation between the iturin A yields and the TPC in fermentation substrates was negative (Figure 6A). In addition, according to statistical analysis, the protein content was another factor affecting iturin A yield. However, the iturin A yields were positively correlated with the protein contents in fermentation substrates (Figure 6B). Direct utilization of these two residues (92011eR and 48424eR) improved RSM conversion as much as 33%, while indirect utilization of residues with extra protein isolation procedure (92011eR-P and 48424eR-P) could increase iturin A production by 70%. The maximum production of iturin A reached 1.95 g/L, which was about 20% higher than the highest yield (1.64 g/L) reported to date (Chen et al., 2019a). Further optimization of the fermentation process such as fed-batch should be used to further improve iturin A yield, which will require further work.

## Conclusion

In this study, we explore a two-step strategy, combining fungal pretreatment followed by extraction of phenolic compounds, that provide a good solution leading to a fully utilization of RSM. This two-step strategy could not only increase extraction efficiency and antioxidant property of phenolic compounds by about 2-fold, but also improve conversion efficiency of remaining meal or protein residue into iturin A production by about 33%. The antioxidant and antibacterial activities of phenolic extracts were influenced by both total phenolic content and profile, while the feed and microbial feedstock value of residue was greatly improved because protein content was increased by ∼5% and antibacterial and anti-nutritional phenolic content was decreased by ∼60%. In addition, this two-step process resulted in the isolation of more proteins from the residue, bringing iturin A production to 1.95 g/L. In conclusion, this two-step strategy is a step forward for the fully utilization of RSM and its potential added value. The method, combining biological pretreatment with chemical extraction and biotransformation, takes the graded utilization of phenolic extract and protein residue into consideration, which accords with the concept of biorefinery.

## Data Availability

The original contributions presented in the study are included in the article/Supplementary Material, further inquiries can be directed to the corresponding author.
