# Supplementary material for: A Two-step Strategy for High-Value-Added Utilization of Rapeseed Meal by Concurrent Improvement of Phenolic Extraction and Protein Conversion for Microbial Iturin A Production
Source: Front Bioeng Biotechnol. 2021 Nov 17;9:735714. doi: 10.3389/fbioe.2021.735714 (PMC8635924; doi:10.3389/fbioe.2021.735714)
Supplement: Supplementary file 1 [file DataSheet1.docx]

**
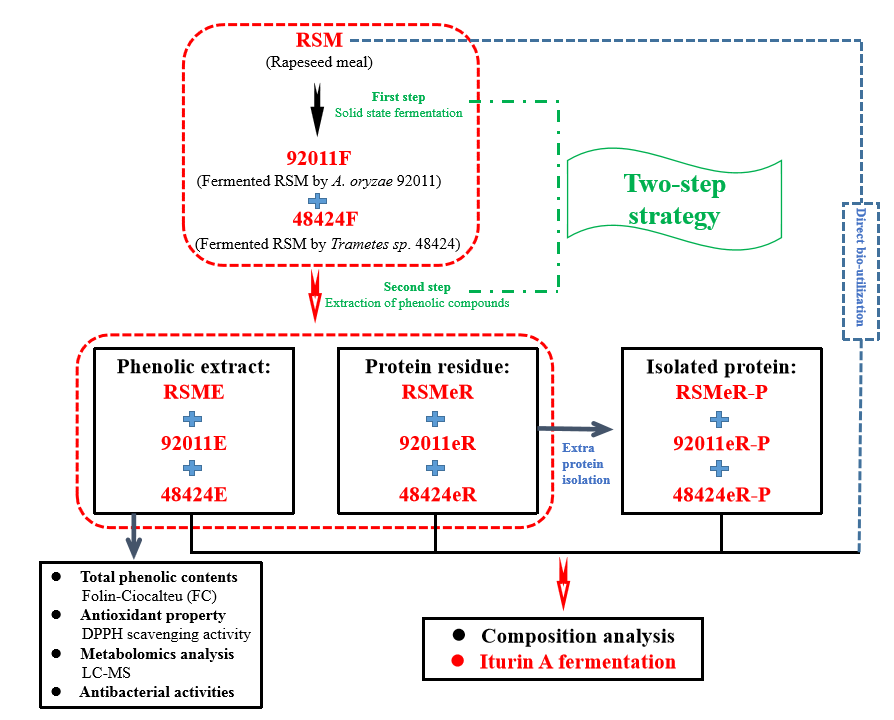
**

**Supplementary Figure 1.** A schematic of the overall experimental procedure.

| **Supplementary Table 1.** Potential flavonoid metabolomics identified among different groups. | | | | | | | | | |
| --- | --- | --- | --- | --- | --- | --- | --- | --- | --- |
|  | Metabolites | Rt (min) | m/z | Score | Fragmentation score | 92011E  compared to RSME | 48424E  compared to RSME | 92011E  compared to 48424E | Pathway |
| 1 | Luteolin | 5.60 | 287.0554 | 40.8 | 11.8 | ↓ | ↑ | ↓ | Flavonoid biosynthesis |
| 2 | Kaempferol 3-O-glucoside | 5.60 | 448.101 | 39.1 | 5.64 | ↓ | ↑ | ↓ | Flavone and flavonol biosynthesis |
| 3 | (+)-Gallocatechin | 4.79 | 306.0744 | 34.1 | 2.25 | ↑ | ↓ | ↑ | Flavonoid biosynthesis |
| 4 | Kaempferol | 6.41 | 287.0552 | 55.2 | 77.8 | ↑ | ↑ | – | Flavonoid biosynthesis |
| 5 | Apigenin 7-O-beta-D-glucoside | 6.51 | 415.1024 | 49.6 | 70.5 | ↑ | ↑ | ↑ | Flavone and flavonol biosynthesis |
| 6 | Rhamnetin | 6.51 | 317.0657 | 50.8 | 57 | ↑ | ↑ | ↓ | NULL |
| 7 | Baicalein 5,6,7-trimethyl ether | 4.66 | 330.1362 | 32.2 | 6.55 | ↑ | ↑ | – | NULL |
| 8 | Proanthocyanidin A2 | 4.21 | 577.139 | 45.4 | 43 | ↑ | ↑ | ↓ | NULL |
| 9 | Malvidin 3-O-glucoside | 4.47 | 494.1391 | 37.6 | 9.07 | ↑ | ↑ | – | Anthocyanin biosynthesis |
| 10 | Phlorizin | 3.86 | 419.1317 | 41.5 | 23.8 | ↑ | ↑ | ↓ | NULL |
| 11 | Delphinidin 3-O-glucoside | 3.99 | 448.0963 | 36.7 | 1.33 | ↑ | ↑ | ↓ | Anthocyanin biosynthesis |
| 12 | Morusin | 4.34 | 443.1477 | 38.8 | 15.6 | ↑ | – | ↑ | NULL |
| 13 | Isoscutellarein | 4.27 | 287.0553 | 41.6 | 11.3 | ↓ | ↓ | ↓ | NULL |
| 14 | Diosmetin | 4.64 | 318.0979 | 37 | 2.81 | ↓ | ↓ | ↓ | NULL |
| 15 | Isorhamnetin | 5.03 | 317.067 | 37.9 | 4.24 | ↓ | ↓ | ↓ | NULL |
| 16 | (-)-Epicatechin | 5.66 | 313.0695 | 41.5 | 21.3 | ↓ | ↓ | ↑ | Flavonoid biosynthesis |
| 17 | 4',5,6,7-Tetramethoxyflavone | 5.16 | 343.1192 | 36.4 | 9.21 | ↓ | ↓ | – | NULL |
| 18 | Hesperetin | 4.51 | 303.0879 | 36 | 11.7 | ↓ | ↓ | ↑ | Flavonoid biosynthesis |
| 19 | Quercetin 3-O-glucoside | 4.40 | 465.1027 | 38.9 | 0.0232 | ↓ | ↓ | ↑ | Flavone and flavonol biosynthesis |
| 20 | Quercetin | 4.14 | 303.0514 | 37.2 | 0.195 | ↓ | ↓ | ↑ | Flavonoid biosynthesis |
| 21 | Luteolin 7-O-beta-D-glucoside | 5.45 | 449.1081 | 44.8 | 26.6 | ↓ | ↓ | ↑ | Flavone and flavonol biosynthesis |
| 22 | Multinoside A | 5.07 | 610.1547 | 39.6 | 5.5 | ↓ | ↓ | ↓ | NULL |
| 23 | Sexangularetin | 4.72 | 317.0659 | 38.2 | 1.67 | ↓ | ↓ | – | NULL |
| 24 | Luteolinidin | 4.79 | 294.0521 | 27.4 | 4.98 | ↓ | ↓ | ↑ | NULL |
| 25 | Rutin | 4.69 | 633.1438 | 39.1 | 0.587 | ↓ | ↓ | ↑ | Flavone and flavonol biosynthesis |
| 26 | Baicalein | 4.79 | 253.0492 | 38.7 | 11.4 | ↓ | ↓ | – | NULL |
| 27 | Tangeretin | 5.07 | 372.1176 | 36.6 | 14.4 | ↓ | ↓ | ↓ | NULL |
| 28 | Cyclomorusin | 5.15 | 436.1716 | 25.7 | 1.55 | ↓ | ↓ | ↓ | NULL |
| 29 | Flavone | 4.82 | 205.0644 | 39.1 | 12.3 | ↓ | ↓ | ↓ | NULL |
| 30 | 4'-Methylcapillarisin | 5.03 | 315.0882 | 37.4 | 2.22 | ↓ | ↓ | – | NULL |
| 31 | 4'-Hydroxy-5,6,7- trimethoxyflavone | 4.79 | 328.0945 | 42.4 | 24.4 | – | ↓ | ↑ | NULL |
| 32 | Sinensetin | 3.69 | 390.1579 | 44 | 55.7 | – | ↓ | ↑ | NULL |
| 33 | (-)-Epiafzelechin | 6.45 | 275.0889 | 38.7 | 14.3 | – | ↓ | ↑ | Flavonoid biosynthesis |
| 34 | Apigenin 7,4'-dimethyl ether | 4.82 | 321.0735 | 38.3 | 8.95 | ↓ | ↓ | ↑ | NULL |
| 35 | Morusinol | 5.50 | 456.2029 | 42.3 | 26.9 | – | ↑ | – | NULL |
| 36 | Nobiletin | 5.00 | 403.1384 | 36.5 | 10.8 | ↓ | ↓ | – | NULL |
| 37 | 5,7-Dimethoxyflavone | 4.77 | 283.0985 | 35.8 | 24.3 | – | – | – | NULL |
| 38 | Pinocembrin | 5.85 | 257.0792 | 45 | 53.3 | ↓ | – | ↓ | Flavonoid biosynthesis |
| 39 | Flavoxate | 5.23 | 374.1744 | 32.5 | 3.97 | ↑ | – | – | NULL |
| 40 | Ginkgetin | 0.69 | 566.1193 | 35.8 | 9.84 | ↑ | ↑ | ↓ | NULL |
| 41 | Norwogonin | 4.81 | 269.0453 | 41 | 20.3 | ↑ | – | ↑ | NULL |
| 42 | Quercitrin | 5.61 | 447.093 | 41.5 | 11.2 | – | ↑ | ↓ | Flavone and flavonol biosynthesis |
| 43 | Naringin | 10.35 | 615.1536 | 37.5 | 5.85 | ↓ | ↓ | – | Flavonoid biosynthesis |
| 44 | Cyanidin 3-O-rutinoside | 9.58 | 594.1646 | 37.4 | 19.3 | ↓ | ↑ | ↓ | Anthocyanin biosynthesis |
| 45 | Naringenin | 6.08 | 271.06 | 49 | 53.6 | ↑ | ↑ | ↓ | Flavonoid biosynthesis |
| 46 | Tricin | 5.96 | 365.0443 | 42.6 | 20.6 | ↑ | – | ↑ | NULL |
| 47 | (+)-Epicatechin | 5.69 | 289.0726 | 39.6 | 4.53 | ↓ | ↓ | ↑ | NULL |
| 48 | Cyanidin 3-O-3'',6''-O-imalonylglucoside; | 5.26 | 620.1023 | 39.6 | 9.11 | ↓ | ↓ | ↓ | Anthocyanin biosynthesis |
| 49 | Cinnamtannin D1 | 5.05 | 899.1611 | 37 | 0.0638 | – | ↑ | – | NULL |
| 50 | Kaempferitrin | 5.00 | 577.1559 | 38 | 3.18 | – | ↓ | ↑ | NULL |

| **Supplementary Table 2****.** Potential cinnamic acids and derivatives metabolomics identified among different groups. | | | | | | | | | |
| --- | --- | --- | --- | --- | --- | --- | --- | --- | --- |
|  | Metabolites | Rt (min) | m/z | Score | Fragmentation score | 92011E  compared to RSME | 48424E  compared to RSME | 92011E  compared to 48424E | Pathway |
| 1 | Sinapic acid | 5.16 | 224.07 | 49.9 | 50.9 | ↑ | – | ↑ | Phenylpropanoid biosynthesis |
| 2 | Sinapine | 4.76 | 328.1972 | 35.7 | 0.307 | ↑ | ↑ | – | Phenylpropanoid biosynthesis |
| 3 | Ferulate | 5.15 | 177.05 | 37.8 | 1.02 | ↓ | ↓ | ↓ | Phenylpropanoid biosynthesis |
| 4 | 5-Hydroxyferulic acid methyl ester | 4.7 | 193.0493 | 39.1 | 2.8 | ↓ | ↓ | – | Phenylpropanoid biosynthesis |
| 5 | cis-2-Hydroxycinnamate | 4.24 | 164.0473 | 47.9 | 42.9 | ↓ | ↓ | – | Phenylpropanoid biosynthesis |
| 6 | p-Coumaroylagmatine | 5.33 | 299.149 | 35.6 | 9.83 | ↓ | – | ↓ | Arginine and proline metabolism |
| 7 | p-Methoxycinnamic acid ethyl ester | 0.69 | 224.128 | 39.1 | 2.5 | – | – | – | NULL |
| 8 | trans-2-Hydroxycinnamate | 3.92 | 165.0537 | 34 | 1.75 | ↑ | – | – | Phenylalanine metabolism |
| 9 | N-Caffeoylputrescine | 4.67 | 250.1322 | 32.2 | 6.23 | ↑ | ↑ | ↑ | NULL |
| 10 | Caffeate | 0.69 | 198.0773 | 48.8 | 74.5 | ↑ | ↑ | – | Phenylpropanoid biosynthesis |
| 11 | Cinnamic acid | 5.2 | 166.086 | 41.7 | 12.1 | ↑ | ↑ | ↓ | NULL |
| 12 | Feruloylputrescine | 4.36 | 265.1544 | 36.3 | 0.361 | ↑ | ↑ | ↑ | Arginine and proline metabolism |
| 13 | Casimiroedine | 0.69 | 440.1782 | 37 | 19.6 | ↑ | ↑ | ↑ | NULL |
| 14 | N1,N8-Bis(4-coumaroyl) spermidine | 4.97 | 436.2220 | 37 | 0.299 | ↓ | ↓ | – | NULL |
| 15 | Feruloylagmatine | 5.92 | 341.1378 | 31.8 | 8.85 | – | – | – | Arginine and proline metabolism |

| **Supplementary Table 3.** Potential coumarins and derivatives metabolomics identified among different groups. | | | | | | | | | |
| --- | --- | --- | --- | --- | --- | --- | --- | --- | --- |
|  | Metabolites | Rt (min) | m/z | Score | Fragmentation score | 92011E  compared to RSME | 48424E  compared to RSME | 92011E  compared to 48424E | Pathway |
| 1 | Scoparone | 4.24 | 207.0653 | 49.5 | 49.4 | ↓ | ↓ | – | NULL |
| 2 | 4-Methylumbelliferyl acetate | 6.13 | 218.0578 | 40.6 | 16.2 | ↓ | ↓ | ↑ | NULL |
| 3 | 4-Methylumbelliferone | 5.86 | 194.0808 | 49.1 | 75.4 | ↓ | ↓ | ↓ | NULL |
| 4 | Benzopyran-3,4-dione | 6.25 | 351.1076 | 40.5 | 23.4 | ↓ | ↓ | – | Metabolism of xenobiotics by cytochrome P450 |
| 5 | Warfarin | 4.84 | 309.1117 | 44.9 | 36.2 | ↓ | ↓ | ↓ | NULL |
| 6 | Scopoletin | 4.24 | 192.0422 | 52.1 | 63.5 | ↓ | ↓ | – | Phenylpropanoid biosynthesis |
| 7 | Coumarin | 5.16 | 146.0364 | 51.5 | 63.1 | ↓ | ↓ | – | Phenylpropanoid biosynthesis |
| 8 | Benzopyran-6-acetaldehyde | 5.63 | 331.0822 | 44.7 | 30.4 | ↓ | ↓ | ↓ | Metabolism of xenobiotics by cytochrome P450 |
| 9 | Fraxidin | 5.02 | 205.0478 | 44.9 | 35.9 | ↓ | ↓ | – | NULL |
| 10 | Herniarin | 4.20 | 177.0544 | 51.7 | 72.2 | – | – | – | NULL |
| 11 | Seselin | 7.57 | 229.0857 | 57.9 | 97.4 | ↓ | ↓ | – | NULL |
| 12 | Dicumarol | 6.92 | 337.0705 | 38.9 | 14.9 | ↑ | – | ↑ | NULL |
| 13 | Osthenol | 4.58 | 269.0596 | 39.6 | 10.8 | ↑ | ↑ | ↑ | Biosynthesis of phenylpropanoids |
| 14 | Heliettin | 3.97 | 337.1412 | 37.5 | 3.95 | ↑ | – | ↑ | NULL |
| 15 | 4-Hydroxycoumarin | 4.48 | 180.0664 | 38.4 | 5.66 | ↑ | ↑ | ↓ | NULL |
| 16 | Novobiocin | 4.34 | 630.2645 | 34.8 | 0.678 | ↑ | ↑ | ↑ | Novobiocin biosynthesis |
| 17 | Esculetin | 4.76 | 177.0182 | 39.5 | 6.13 | ↓ | ↓ | ↑ | Biosynthesis of phenylpropanoids |
| 18 | Alternariol | 5.23 | 257.0479 | 35.2 | 10.1 | ↑ | ↓ | ↑ | NULL |
| 19 | Umbelliferone | 5.00 | 161.0229 | 41.5 | 25.5 | ↑ | ↓ | ↑ | Biosynthesis of phenylpropanoids |

| **Supplementary Table 4.** Potential isoflavonoids metabolomics identified among different groups. | | | | | | | | | |
| --- | --- | --- | --- | --- | --- | --- | --- | --- | --- |
|  | Metabolites | Rt (min) | m/z | Score | Fragmentation score | 92011E  compared to RSME | 48424E  compared to RSME | 92011E  compared to 48424E | Pathway |
| 1 | 2'-Hydroxybiochanin A | 4.64 | 318.0979 | 36.9 | 2.71 | ↓ | ↓ | ↓ | Isoflavonoid biosynthesis |
| 2 | Cajanin | 4.97 | 318.097 | 37.5 | 2.12 | ↓ | – | ↓ | NULL |
| 3 | Ferreirin | 4.51 | 303.0879 | 35.1 | 7.21 | ↓ | ↓ | ↑ | NULL |
| 4 | Genistein | 4.81 | 269.0453 | 38.7 | 8.74 | ↑ | – | ↑ | Isoflavonoid biosynthesis |
| 5 | Dihydrobiochanin A | 4.79 | 309.0753 | 35.8 | 24.3 | ↓ | ↓ | – | Isoflavonoid biosynthesis |
| 6 | Licoisoflavone A | 5.66 | 355.1183 | 36.1 | 6.65 | ↓ | ↓ | ↑ | NULL |
| 7 | Irisolidone | 5.03 | 315.0882 | 37 | 0.0882 | ↓ | ↓ | – | NULL |
| 8 | Afrormosin | 4.82 | 321.0735 | 38 | 7.65 | ↓ | ↓ | ↑ | NULL |
| 9 | Sojagol | 6.13 | 337.1074 | 29.1 | 3.19 | ↓ | ↓ | ↓ | NULL |
| 10 | Kievitone hydrate | 6.29 | 375.1451 | 40.7 | 39.8 | – | – | – | NULL |
| 11 | Dihydrogenistein | 5.26 | 290.1028 | 33.5 | 2.31 | ↓ | – | ↓ | NULL |
| 12 | Cyclokievitone | 5.1 | 337.1078 | 34.6 | 10.2 | ↓ | ↓ | ↓ | NULL |
| 13 | Kievitone | 4.79 | 357.1337 | 39.3 | 20.7 | ↑ | – | ↑ | NULL |
| 14 | 5-Deoxykievitone | 3.86 | 363.1223 | 36.8 | 0.504 | ↑ | ↑ | ↑ | NULL |
| 15 | 2'-Hydroxygenistein | 6.41 | 287.06 | 55.2 | 77.8 | ↑ | ↑ | – | Isoflavonoid biosynthesis |
| 16 | Glycyrol | 0.66 | 367.1193 | 38.6 | 6.79 | ↑ | ↑ | ↑ | NULL |
| 17 | Rotenone | 0.69 | 412.18 | 38.6 | 5.93 | ↑ | ↑ | ↑ | Isoflavonoid biosynthesis |
| 18 | Daidzin | 6.49 | 416.1107 | 52.9 | 67.2 | ↑ | – | ↑ | Isoflavonoid biosynthesis |
| 19 | Malonylglycitin | 4.54 | 567.0862 | 35.4 | 2.24 | ↓ | – | ↓ | Isoflavonoid biosynthesis |
| 20 | Pseudobaptigenin | 4.79 | 281.0433 | 37.1 | 5.82 | ↑ | ↓ | ↑ | Isoflavonoid biosynthesis |
| 21 | Cajanol | 6.17 | 315.0852 | 39.1 | 7.45 | – | – | – | NULL |
| 22 | Irilone | 4.81 | 297.0392 | 36.4 | 3.06 | ↑ | ↑ | ↑ | NULL |

| **Supplementary Table 5.** Potential stilbenes metabolomics identified among different groups. | | | | | | | | | |
| --- | --- | --- | --- | --- | --- | --- | --- | --- | --- |
|  | Metabolites | Rt (min) | m/z | Score | Fragmentation score | 92011E  compared to RSME | 48424E  compared to RSME | 92011E  compared to 48424E | Pathway |
| 1 | Piceid | 5.09 | 413.1217 | 39 | 5.46 | ↓ | – | ↓ | NULL |
| 2 | Demethylbatatasin IV | 4.58 | 269.0596 | 38.6 | 5.86 | ↑ | ↑ | ↑ | NULL |
| 3 | Batatasin IV | 4.55 | 267.0975 | 36.2 | 2.11 | ↑ | – | ↑ | NULL |
| 4 | Toremifene | 3.99 | 388.1827 | 32.1 | 5.57 | ↑ | ↑ | ↑ | NULL |
| 5 | Narceine | 0.69 | 446.1830 | 31 | 7.41 | ↑ | ↑ | ↑ | NULL |
| 6 | Hydroxytamoxifen | 4.04 | 422.1931 | 38.8 | 11.5 | ↑ | ↑ | ↑ | Drug metabolism- cytochrome P450 |
| 7 | 3-Hydroxy-5-methoxy-6-prenylstilbene-2-carboxylic acid | 4.00 | 373.1228 | 30.2 | 30.4 | ↑ | – | ↑ | NULL |
